# GIS-based spatiotemporal mapping of malaria prevalence and exploration of environmental inequalities

**DOI:** 10.1007/s00436-024-08276-0

**Published:** 2024-07-06

**Authors:** Ropo Ebenezer Ogunsakin, Bayowa Teniola Babalola, Johnson Adedeji Olusola, Ayodele Oluwasola Joshua, Moses Okpeku

**Affiliations:** 1 School of Health Systems and Public Health, University of Pretoria Faculty of Health Sciences, Pretoria, South Africa; 2 Department of Statistics, Ekiti State University, Ado Ekiti, Nigeria; 3 Department of Geography and Planning Science, Ekiti State University, Ado Ekiti, Nigeria; 4 Department of Mathematical Sciences, Science and Technology, Bamidele Olumilua University of Education, Ikere Ekiti, Nigeria; 5 Discipline of Genetics, School of Life Sciences, University of Kwa-Zulu Natal, Westville, Durban, South Africa

**Keywords:** Malaria, Environmental indicators, Geographic information systems, Spatial pattern analysis, Spatial statistics analysis, Spearman correlation, Ordinary least squares, Demographic and health surveys

## Abstract

Malaria poses a significant threat to global health, with particular severity in Nigeria. Understanding key factors influencing health outcomes is crucial for addressing health disparities. Disease mapping plays a vital role in assessing the geographical distribution of diseases and has been instrumental in epidemiological research. By delving into the spatiotemporal dynamics of malaria trends, valuable insights can be gained into population dynamics, leading to more informed spatial management decisions. This study focused on examining the evolution of malaria in Nigeria over twenty years (2000–2020) and exploring the impact of environmental factors on this variation. A 5-year-period raster map was developed using malaria indicator survey data for Nigeria’s six geopolitical zones. Various spatial analysis techniques, such as point density, spatial autocorrelation, and hotspot analysis, were employed to analyze spatial patterns. Additionally, statistical methods, including Principal Component Analysis, Spearman correlation, and Ordinary Least Squares (OLS) regression, were used to investigate relationships between indicators and develop a predictive model. The study revealed regional variations in malaria prevalence over time, with the highest number of cases concentrated in northern Nigeria. The raster map illustrated a shift in the distribution of malaria cases over the five years. Environmental factors such as the Enhanced Vegetation Index, annual land surface temperature, and precipitation exhibited a strong positive association with malaria cases in the OLS model. Conversely, insecticide-treated bed net coverage and mean temperature negatively correlated with malaria cases in the same model. The findings from this research provide valuable insights into the spatiotemporal patterns of malaria in Nigeria and highlight the significant role of environmental drivers in influencing disease transmission. This scientific knowledge can inform policymakers and aid in developing targeted interventions to combat malaria effectively.

## Background

Malaria, a significant global health threat, causes extensive illness and death worldwide, with a particularly severe impact on the African continent (Sankineni et al. 2023; Simon-Oke et al. 2023; Rodríguez et al. 2023). This disease has posed a persistent challenge to public health on a global scale for many years, especially in tropical regions. The primary Plasmodium species responsible for malaria are *Plasmodium falciparum*, *Plasmodium vivax*, *Plasmodium ovale*, and *Plasmodium malaria,* with *P. falciparum* being the deadliest and accounting for up to 95% of malaria implicated deaths in Africa (Sankineni et al. 2023; Simon-Oke et al. 2023). Despite a modest decrease in malaria prevalence in recent years, as indicated by past research, many individuals globally, particularly in Africa, continue to grapple with malaria due to insufficient socio-economic status and access to treatment resources. (Das et al. 2023; Mac et al. 2023; Sarfo et al. 2023; Taiwo et al. 2023).

Similarly, the World Malaria Report highlighted a staggering 247 million malaria cases in 2021, resulting in over 600,000 fatalities during the same year (World Health Organization 2023), with only four African countries accounting for more than half of all malaria deaths worldwide; Nigeria alone accounting for about 31.3%. These statistics made Nigeria the leading country with the highest proportion of malaria deaths in 2021. Despite numerous studies on malaria prevention in Nigeria, high malaria prevalence persists in many regions (Kanmiki et al. 2019). Strengthening prevention efforts remains crucial. Understanding transmission variations can aid in developing effective control strategies and resource allocation.

Despite government efforts at different levels to combat malaria, high prevalence persists in regions with heavy rainfall and warm temperatures (Oviedo et al. 2023). Rainfall creates breeding sites for mosquitoes, the vectors of malaria, while warmer temperatures accelerate the malaria parasite’s growth and prolong the mosquito’s lifespan. Consequently, malaria is particularly prevalent in areas with heavy rainfall and warm temperatures. Malaria prevalence in Nigeria differs across geopolitical regions because of the varying environmental and seasonal settings that affect the reproductive patterns of mosquito vectors. The prevalence of malaria is primarily a function of its underlying transmission intensity (Alegana et al. 2013), which in turn is propelled by indicators such as interventions (García et al. 2023), environmental and climatic factors (Ekpa et al. 2023; Rivera and Gutiérrez 2023), and socio-economic and demographic characteristics (Ogunsakin and Chen 2020; Pourtois et al. 2023; Rivera and Gutiérrez 2023). In malaria prevalence research, authors have linked malaria rates to environmental and socio-economic factors like population density and potential evapotranspiration (PET) (Yang et al. 2012, 2005). Since the indicators affecting malaria are diverse, those considered in this study were selected based on previous studies, as acknowledged above. Hence, using malaria indicator survey data, the current research employed time-based spatial mapping of *the* prevalence of *Plasmodium falciparum* malaria.

Besides, these cross-sectional surveys are aimed at being comprehensive and nationally representative, where information on several indicators affecting the prevalence of *Plasmodium falciparum* is gathered. The essence of this survey is to provide recent estimates of fundamental demographic-related and health-related malaria indicators. It is intended to provide estimates at the national level, as well as in urban and rural areas and six geopolitical regions. They are conducted with a standardized methodology. Unlike other data types, nationally representative cross-sectional data is invulnerable to incompleteness or standards for clinical diagnosis (Arambepola et al. 2020; Uwemedimo et al. 2018). This explains why prevalence information from national health surveys is the primary source of data for mapping malaria risk in many countries has always been, particularly in Africa (Weiss et al. 2019; Battle et al. 2019).

This study aimed to determine the spatiotemporal trends of malaria distribution in Nigeria and to determine potential relationships between malaria prevalence and environmental indicators. Disease mapping is widely used in public health surveillance since it describes the spatiotemporal variation of the disease, identifies areas with unusually high risk, and formulates etiological hypotheses (Lawson 2018). Therefore, probing the spatiotemporal dynamics of malaria by linking GPS data to external covariates can lead to new intuitions in population processes and foster a track toward enhanced spatial management decisions. Environmental indicators were selected to establish a spatiotemporal distribution model using a geographical information system (GIS). GIS has proven to be a powerful technique for public health surveillance across various geographical areas (Musa et al. 2013; Kamel Boulos and Geraghty 2020).

## Method

### Data

**Fig. 1:**
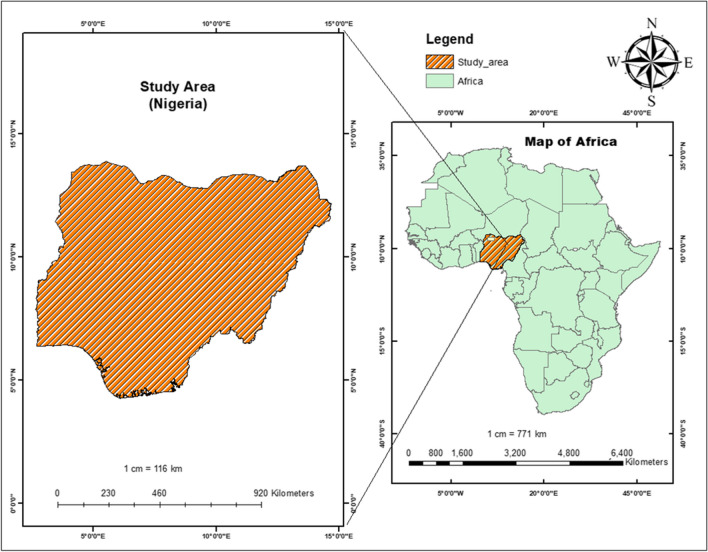
Geographical setting of the study area mapping

The study was conducted in Nigeria, a sub-Saharan African country, between latitudes 4º16’ and 13º53’ N and longitudes 2º40’ and 14º41’ E. It shares borders with the Niger Republic in the north, the Republic of Chad in the northeast, the Republic of Cameroon in the east, and the Republic of Benin in the west. The climate and topography of Nigeria are diverse, encompassing highlands (600 to 1300 m in the North Central Zone), eastern highlands, and lowlands (less than 20 m in coastal areas). Two seasons in one year are wet and dry. The dry season runs from October to March, with a wave of freshness accompanied by the dry and dusty wind of Harmattan, mainly experienced in the north in December and January. The wet season begins in April and ends in September. The country is divided into 36 states and a Federal Capital Territory [FCT], consisting of six geopolitical zones (Fig. 1), and covers an area of about 923,769 square kilometres. The states are categorized into six geopolitical zones with 774 constitutionally recognized local government areas (LGAs). The country has a total surface area of approximately 910,770 square kilometers (351,650 square miles) and a population density of 246 per Km^2^ (636 people per mi^2^) (https://www.worldometers.info/world-population/nigeria-population/). About 53.8 percent lived in urban centres, while 46.2 percent lived in rural areas (https://countrymeters.info/en/Nigeria). Administrative boundaries were also consulted through the Demographic and Health Survey (DHS) Spatial Data Repository (https://spatialdata.dhsprogram.com/). Administrative boundaries are subnational regions, usually administrative level 1, and vary between survey years and countries.

### Sampling procedures

The 2021 Nigeria Malaria Indicator Surveys (NMIS), made available on the DHS Program website, were used in this study. The DHS is the primary source of benchmarking information on women’s and young children’s health in most developing countries. It has been established that it is also helpful in investigating the connection between environmental factors and health (Boyle et al. 2020). The 2021 NMIS utilized a two-staged sampling procedure. In the first stage, 568 enumeration areas (EAs) were chosen with probability proportional to the EA size. In the second stage, 25 households per urban and 25 per rural cluster were selected. The size of the EA is the number of families resident in the EA. The selection of the sample was carried out to be representative of each state. This resulted in the selection of 568 clusters in the country: 195 in urban areas and 373 in rural areas. The geospatial covariates of 2021 NMIS used for the study contained data on malaria prevalence and environmental indicators. This geospatial data was measured with remote sensing within two kilometres in urban areas and 10 kms in rural areas around the site of the DHS survey cluster for 568 clusters across the country for five years (2000, 2005, 2010, 2015, and 2020).

### Data sources for malaria prevalence

The Demography and Health Survey remains a valuable source that, combined with complementary information, may provide the evidence base to understand better human health resources and resource allocation (Ogunsakin and Ginindza 2022). This study uses secondary DHS data and is available upon request for download from the DHS Website. Geospatial covariate data from DHS and Geographic Position System (GPS) cluster points were obtained from the GDPS spatial repository. DHS spatial cluster data (*n* = 568) are GPS points captured with survey data. Most DHS surveys are now geocoded, whereas a GPS coordinate is recorded in the approximate centre of each primary sampling unit. To keep participants confidential, GPS coordinates were moved to protect the confidentiality of participants by grouping households into groups and replacing them by up to 0–2 kms for urban areas and 0–10 kms for rural regions (Bennett and Smith 2017), geospatial covariates often accompany the data, and it is frequently challenging to link the data with the DHS programmer’s data to determine the impact of location on health outcomes. To mitigate this challenge, the DHS geospatial team developed a set of standard geospatial covariate files that have already been used for the dataset. The covariate indicators were obtained from raster and vector data. Raster data, like images and patterned areas, are based on pixels or cells to transmit data values. On the other hand, vector data, such as dots, lines, and polygons, show a characteristic’s location or discrete limit. A full description of the DHS geospatial covariate dataset and methodology is available (Bennett and Smith 2017).

### Data collection and preparation

Environmental indicators (Table 1**)** related to malaria prevalence were compiled from the Nigeria Demographic Health Survey (NDHS). They included the Aridity Index (AI), Enhanced Vegetation Index (EVI), insecticide-treated bed net (ITN) coverage, Maximum Temperature (MT), precipitation, rainfall, daytime and night-time land surface temperatures, and Mean annual Potential Evapotranspiration (PET). Additionally, Nigeria shape files from DIVAS-GIS (https://www.diva-gis.org/gdata) were utilized. These indicators were selected to establish a spatiotemporal distribution model and use a Geographical Information System (GIS). These data often come with geospatial covariates, and it is frequently challenging to link them with the DHS Programmer’s data to determine the impact of location on health outcomes. To alleviate the difficulty, the DHS Programme Geospatial Team developed a set of standardized files of the most used geospatial covariates already linked with the dataset. The prevalence of malaria was measured in the NDHS using the average *Plasmodium falciparum* parasite rate (PfPR). This study obtained the DHS malaria survey year from the DHS spatial data repository site (https://spatialdata.dhsprogram.com/). The prevalence of malaria depends first and foremost on its underlying transmission intensity (Alegana et al. 2013), which is driven by indicators such as interventions and environmental/climatic, socio-economic, and demographic factors.

**Table 1:** Spatial and temporal resolution of geospatial datasets covariates included in the model

Geospatial dataset	Descriptions	Units	Spatial resolution	Temporal resolution	Source	Format	Type	Covariate Spatial detail	Temporal coverage
Aridity	Mean annual aridity	%	−	−	−	Raster	Continuous	DHS sub- national area	2000–2020
EVI	Enhanced vegetation index	Index	1km	Monthly	MODIS	Raster	Continuous	DHS sub- national area	2000–2020
LST	Annual Land Surface Temperature	^0^C	1km	1 day	MODIS	Raster	Continuous	DHS sub- national area	2000–2020
ITNC	Insecticide-treated bed nets coverage	Proportion of the population	−	−	−	Raster	Continuous	DHS sub- national area	2000–2020
MT	Maximum Temperature	^0^C	1km	Monthly	MODIS	Raster	Continuous	DHS sub- national area	2000–2020
Precipitation	Average Precipitation	Millimeters	5km	Monthly	CHIRPS	Raster		DHS sub- national area	2000–2020
Rainfall	Climate hazards group infrared precipitation with station data	Millimeters	5km	Monthly	CHIRPS	Raster	Continuous	DHS sub- national area	2000–2020
LSTday	Land surface temperature in the daytime	^0^C	1km	Monthly	MODIS	Raster	Continuous	DHS sub- national area	2000–2020
LSTnight	Land surface temperature in the night-time	^0^C	1km	Monthly	MODIS	Raster	Continuous	DHS sub- national area	2000–2020
PET	Mean annual Potential Evapotranspiration	Millimeters	1km	Monthly	MODIS	Raster	Continuous	DHS sub- national area	2000–2020
Wet days		Millimeters	1km	Monthly	MODIS	Raster	Continuous	DHS sub- national area	2000–2020

*MODIS* moderate resolution imaging spectroradiometer, *CHIRPS* climate hazards group infrared precipitation with station data

### Statistical analysis and spatial analysis models

To increase our insight into how malaria spreads through space and how several host compartments are linked, comprehensive information was obtained from 2021 Malaria Indicator Surveys (MIS). This type of survey provides another data source for understanding the spatiotemporal trends of malaria endemicity. This study described malaria prevalence in different regions in Nigeria from years 2000 to 2020. Descriptive statistical analysis, mean difference, and the association between malaria prevalence and environmental indicators were computed using absolute and relative frequencies and Pearson correlation coefficients.

In the first stage, Kaiser–Meyer–Olkin (KMO)-Bartlett assays were performed to determine the adequacy of spatial analysis data. Initial results of the KMO sampling adequacy measurement indicated that Principal Component Analysis (PCA) would be a suitable statistic for data reduction (Farzinpour et al. 2023). PCA is a linear statistical process universally deployed to reduce data dimensions by extracting the most significant variations from the original data sets (Ocampo-Marulanda et al. 2022). It uses an orthogonal transformation to convert possibly correlated indicators into several linearly uncorrelated, independent PCs. Different co-factors affected by collinearity affect malaria transmission at various stages. PCA allowed for keeping the main environmental features without losing part of the environmental co-factors associated with malaria prevalence. Hence, a PCA with varimax rotation was applied to find this study’s explanatory indicators.

In the second stage, we performed ordinary least square (OLS) regression to test the assumption of the models according to OLS requirements. The OLS is a global model and assumes the variable relationship to be persistent throughout the study area. The ultimate assumption of a multivariate regression model is that the relation between dependent and explanatory variables is spatially constant (Yue et al. 2018; Mohidem et al. 2021). Although the OLS model is not regarded as the best technique for the statistical analysis of spatial data, it has unswervingly been the appropriate initial point for any spatial regression analyses to uncover the significant indicators (explanatory indicators) associated with the outcome variable (malaria prevalence). This model would explain if malaria hotspots occurred due to the combination of these explanatory indicators. The implication of such is that it would assist in creating a prediction map that can be used for public health resource allocations due to the spatial relationship between the dependent and explanatory indicators.

In addition, before the main spatial mapping, the purpose was to pre-process data further and assess the extent of the statistical significance between the outcome and the selected explanatory indicators from the PCA result. In this case, the independent variables were tested for normality using the Shapiro–Wilk test at 0.001. Since the *p*-values were all greater than 0.001, it showed that the independent variables were normally distributed. Using all normally distributed variables, we conducted the regression analysis and developed a model for malaria prevalence. Using this model, we generated a model prevalence raster map via ArcMap’s map calculation functions and complementary datasets. Further, Variance inflation factors (VIFs) were applied to monitor multicollinearity among the indicators through a VIF function in the R statistical programming environment using the “olsrr” package. We observed that multicollinearity does not occur as all the VIF values are less than ten, and the tolerance value is higher than 0.1.

Finally, the global spatial autocorrelation was evaluated using the Global Moran’s I statistic (Moran’s I) to assess the presence of geographical clustering and variability. A positive value for Moran’s Index implies a geographical clustering for malaria. In contrast, a negative value for Moran’s Index implies a dispersion and a zero value is distributed randomly when Moran’s Index is statistically significant. The local Getis-Ord spatial statistical tool was employed to detect statically significant hotspot and cold spot regions. Hotspot refers to the occurrence of high prevalence of malaria clustered together on the map; however, cold spots refer to the occurrence of low prevalence of malaria clustered together on the map. A point density map was applied to examine the temporal pattern of malaria prevalence. The point density tool in ArcMap was used to create the point density map. The point density tool calculates the density of point features around each output raster cell. Detailed maps were built with spatial data to visualize the distribution of malaria prevalence and hot spot analysis. The PCA was performed using the FactoMineR and factoextra packages in the R project. All the spatial maps were produced in ArcMap (version 10.4).

The health sciences database offers a wide array of data, ranging in diversity, size, and complexity, which can be effectively analyzed utilizing Geographic Information System (GIS) tools. Spatial analysis techniques, such as point density, spatial autocorrelation, raster map analysis, and hot spot analysis, were applied to examine spatiotemporal patterns of malaria prevalence. Gaining a deeper understanding of malaria prevalence, particularly spatial patterns, is crucial for allocating resources effectively for malaria prevention and control efforts (Lai et al. 2015).

## Results

### Spatiotemporal statistics of environmental indicators and malaria mean separation across five years

The trends and spatial variation of the environmental indicators from 2000 to 2020 in Nigeria were evaluated in this study. Table 2 provides descriptive characteristics of the study population. This resulted in 568 clusters in the country, 195 in urban areas, and 372 in rural areas. The 2021 NMIS geospatial covariates contained malaria prevalence information. Concerning the data set, we have 2,835 cases of malaria. Table 2 also shows the descriptive trend of the explanatory indicators utilized. As shown, malaria prevalence in Nigeria was 48%, 43%, 35%, 23%, and 27% in 2000, 2005, 2010, 2015, and 2020, respectively.

**Table 2:** Temporal descriptive statistics of explanatory covariates from 2000 to 2020

Environmental factors		Mean ± SD	Minimum	P_25_	Median	P_75_	Maximum
2000	Prevalence	0.48 ± 0.18	0.01	0.36	0.46	0.59	0.93
	Aridity	31.54 ± 18.29	3.75	14.42	29.28	47.85	71.52
	EVI	0.33 ± 0.09	0.12	0.24	0.33	0.41	0.53
	ITNC	0.06 ± 0.01	0.03	0.05	0.06	0.07	0.11
	LST	25.65 ± 1.80	21.33	24.23	25.43	27.13	29.99
	Maximum temperature	32.54 ± 1.39	28.99	31.54	32.00	33.47	36.44
	Mean temperature	26.81 ± 0.82	23.04	26.53	26.84	27.18	28.83
	Precipitation	112.70 ± 44.07	25.65	76.87	105.55	156.13	202.46
2005	Prevalence	0.44 ± 0.18	0.05	0.30	0.42	0.55	0.91
	Aridity	31.31 ± 16.72	6.22	16.42	28.78	44.97	65.18
	EVI	0.32 ± 0.09	0.12	0.24	0.32	0.40	0.51
	ITNC	0.27 ± 0.18	0.01	0.02	0.03	0.03	0.04
	LST	25.66 ± 1.53	20.93	24.29	25.69	26.94	29.12
	Maximum temperature	32.75 ± 1.70	29.32	31.42	32.06	34.13	37.27
	Mean temperature	27.41 ± 0.87	23.80	26.97	27.27	27.79	29.71
	Precipitation	111.21 ± 36.62	42.58	82.44	104.54	140.66	186.84
2010	Prevalence	0.36 ± 0.14	0.07	0.25	0.34	0.44	0.75
	Aridity	34.58 ± 19.80	5.59	15.44	33.48	49.39	71.59
	EVI	0.32 ± 0.09	0.10	0.24	0.32	0.40	0.52
	ITNC	0.18 ± 0.12	0.02	0.08	0.14	0.28	0.52
	LST	25.95 ± 1.57	21.67	24.56	25.86	27.13	29.52
	Maximum temperature	33.08 ± 1.72	29.30	31.72	32.37	34.44	37.52
	Mean temperature	27.67 ± 0.86	23.94	27.21	27.51	28.14	29.79
	Precipitation	124.43 ± 46.69	41.31	82.91	120.60	162.66	215.87
2015	Prevalence	0.23 ± 0.14	0.03	0.13	0.20	0.30	0.74
	Aridity	27.52 ± 16.52	4.31	11.97	26.01	40.43	61.38
	EVI	0.31 ± 0.10	0.09	0.23	0.31	0.40	0.54
	ITNC	0.30 ± 0.16	0.01	0.17	0.27	0.43	0.71
	LST	26.14 ± 1.61	21.38	24.75	26.05	27.49	29.90
	Maximum temperature	32.73 ± 1.45	29.13	31.18	32.18	33.80	36.69
	Mean temperature	27.29 ± 0.84	23.57	26.94	27.22	27.72	29.41
	Precipitation	100.31 ± 42.22	29.44	62.63	92.54	135.34	186.26
2020	Prevalence	0.27 ± 0.13	0.05	0.18	0.26	0.34	0.69
	Aridity	33.17 ± 18.47	6.37	17.11	28.45	48.99	72.53
	EVI	0.31 ± 0.09	0.12	0.23	0.30	0.37	0.52
	ITNC	0.38 ± 0.20	0.06	0.23	0.34	0.52	0.93
	LST	26.68 ± 1.46	21.95	25.47	26.77	27.74	30.20
	Maximum temperature	32.70 ± 1.39	29.21	31.70	32.19	33.73	36.27
	Mean temperature	27.32 ± 0.83	23.53	27.03	27.31	27.68	29.45
	Precipitation	120.03 ± 42.93	42.93	90.50	102.08	159.16	211.59

Malaria prevalence in the six regions of Nigeria between 2000–2020 is summarized in Table 3. Malaria cases were concentrated in the northwest, northcentral, south-south, and Southwest, accounting for 71.3% of all cases. Also, malaria cases were concentrated in rural settings, accounting for 65.6% of the entire cases in the study area. At the regional level, over the five years, malaria cases reached the ultimate number in the Northwest region with 560 cases (19.75%), followed by the North-Central zone with 505 cases, South-South with 495 cases, and Southwest with 460 cases (Table 3).

**Table 3:** Cases of malaria (cases per 1000 population) in the study area, 2000–2020

	Geopolitical zone						Residence	
	North Central	North-East	Northwest	South-East	South-South	Southwest	Rural	Urban
Number	505	445	560	370	495	460	1860	975
Percentage	17.81	15.70	19.75	13.05	17.46	16.23	65.61	34.39

### Multivariate data analysis of environmental indicators

**Fig. 2:**
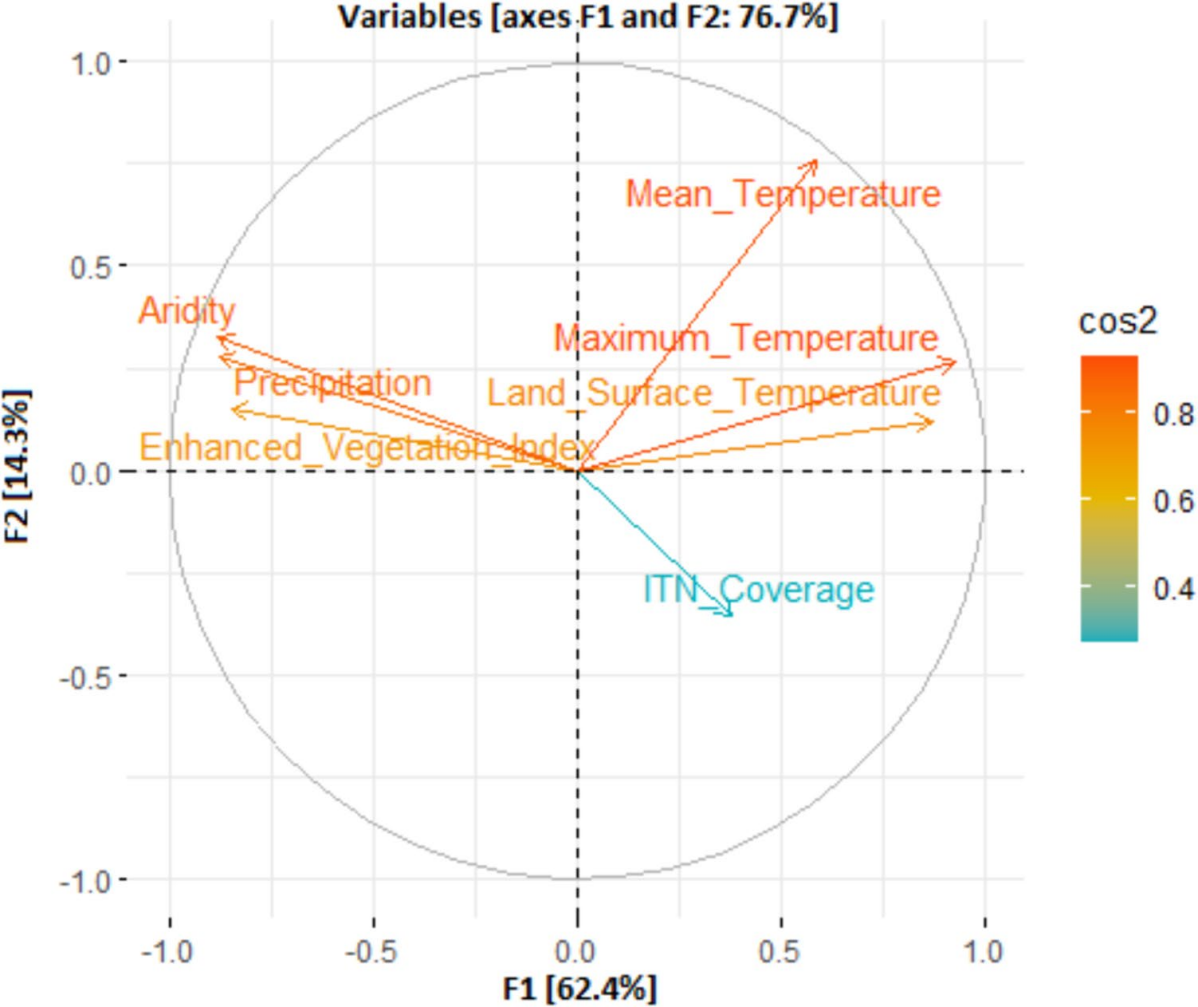
Circle of correlations and plot of the factor loadings of the indicators with F1 and F2

The KMO measure employed for the environmental indicators set with seven indicators is 0.796. Since the KMO test is > 0.50, the environmental indicators set are acceptable for PCA. Bartlett’s test of sphericity has these values: χ^2^ = 22,953.58, degrees of freedom = 21, and *p* < 0.0001 for *α* = 0.05, which is good and indicates that we can proceed with the PCA. According to the empirical rule and the eigenvalues chart, two principal components (PCs) were chosen. The first and second main components explained 62.4% and 14.3%, respectively. Further, the PCA conducted using Kaiser’s criterion resulted in maintaining two environmental indicators that explained 76.7% of the total inertia (Fig. 2). Figure 2 shows the indicator factorial load chart on components and the relationship between indicators in three different ways. Each indicator is a point for which the loads on the PCs give the coordinates. If an indicator is well represented by only two principal components (F1 and F2), the sum of the cos2 on these two PCs equals one. If so, the indicators will be placed on the circle of correlations. The cos2 values serve to estimate the quality of the representation (Mebatsion et al. 2012). The nearer an indicator approaches the circle of correlations, the better its depiction on the factor map (and the greater the importance of interpreting these components). Indicators closed at the trace’s center are less critical for the first components. Additionally, the first principal component explained 62.4% of the total inertia. The indicators that best contributed to this were maximum temperature (13.76%, correlation coefficient r = 0.92), LST (13.40%, r = 0.87), mean temperature (13.08%, r = -0.38), and maximum temperature (10.77%; r = 0.38). The second principal component explained 14.3% of the total inertia. The indicator with the most contribution is the mean temperature (10.08%, r = 0.76).

Based on the Pearson correlation coefficient, the aridity (coefficient = 0.165, *p* =  < 0.001), EVI (coefficient = 0.296, *p* =  < 0.001), and precipitation (coefficient = 0.157, *p* =  < 0.001) indicators explained a positive association with malaria prevalence. In contrast, ITNC (coefficient = -0.377, *p* =  < 0.001), land surface temperature (coefficient = -0.243, *p* =  < 0.001), maximum temperature (coefficient = -0.144, *p* =  < 0.001), and mean temperature (coefficient = -0.171, *p* =  < 0.001) explained a negative association with malaria prevalence. These findings imply some environmental indicators show a direct relationship while others indicate an indirect one.

### Environmental indicators affecting malaria prevalence using the OLS model

Following the results of the correlation analysis, collinearity was carried out among the selected indicators. Since the study environmental indicators do not have a normal distribution according to the Shapiro–Wilk test (Ho: The variable is normally distributed), VIF and conditional index (CI) were calculated for the multicollinearity analysis. However, all the VIFs of the reported indicators are less than 10, indicating no collinearity. The result of the collinearity reveals Average temperature (VIF = 1.479, tolerance = 0.676); precipitation (VIF = 1.947, tolerance = 0.513); ITN Coverage (VIF = 1.122, tolerance = 0.891); enhanced vegetation index (VIF = 3.135, tolerance = 0.319) and land surface temperature (VIF = 3.578, tolerance = 0.279). Hence, the OLS model was fitted to assess the contribution of each essential indicator of malaria prevalence. The OLS model suggests that the indicators have some impact on the study area (Table 4). Besides, the OLS model explains the 44.2% variation in malaria prevalence by environmental indicators. This implies that unknown environmental indicators cause 55.8% of malaria prevalence. The regression coefficients for indicators significantly correlated with malaria are presented in Table 4. It showed the regression coefficients and the robust standard error estimated by the model.

**Table 4:** Effect of Environmental Indicators affecting malaria prevalence in the study area

Indicator	Coefficient [a]	StdError	t-Statistic	Probability [b]	Robust SE	Robust_t	Robust Pr [b]	VIF [c]
Intercept	0.640	0.116	5.501	< 0.001	0.108	5.923	< 0.001	−
EVI	0.622	0.059	10.484	< 0.001	0.057	10.849	< 0.001	3.135
ITN Coverage	−0.319	0.017	−18.357	< 0.001	0.014	−23.606	< 0.001	1.122
LST	0.012	0.004	3.282	< 0.001	0.003	3.654	< 0.001	3.578
Mean temp	−0.025	0.004	−5.862	< 0.001	0.004	−6.154	< 0.001	1.479
Precipitation	0.000	0.000	−4.425	< 0.001	0.00009	−4.493	< 0.001	1.947

### Spatiotemporal trend of malaria prevalence rates from 2000 to 2020

**Fig. 3:**
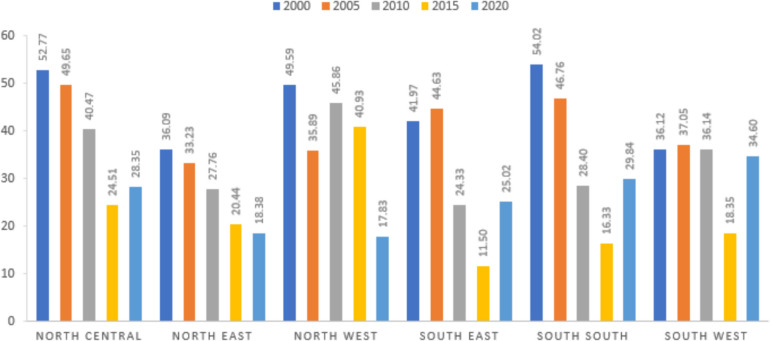
Temporal trend analysis of malaria prevalence in Nigeria between 2000 and 2020

Figure 3 depicts the overall temporal trend of malaria between 2000 and 2020. The South-South took the lead in 2000, followed by the Northcentral and Northwest. Malaria cases decreased significantly among these three geopolitical zones, apart from the northwest, between 2000 and 2020. Over the past year, a substantial number of malaria cases have been detected in the Southwest, which could be attributed to the dense vegetation of the rural area, lack of access to adequate medical facilities, or an unsafe environment (Ekpa et al. 2023). Malaria prevalence has declined in the Northeast year after year from 2000 to 2020**.** The decline in this region could be attributed to a recent government effort to access good health institutions, attempts by residents to make their surroundings hygienic, or appropriate civic education about the impact of illness **(**Oyibo et al. 2021). In general, the overall prevalence of malaria over the five years had a declining trend but with inconsistencies (Fig. 3).

**Fig. 4:**
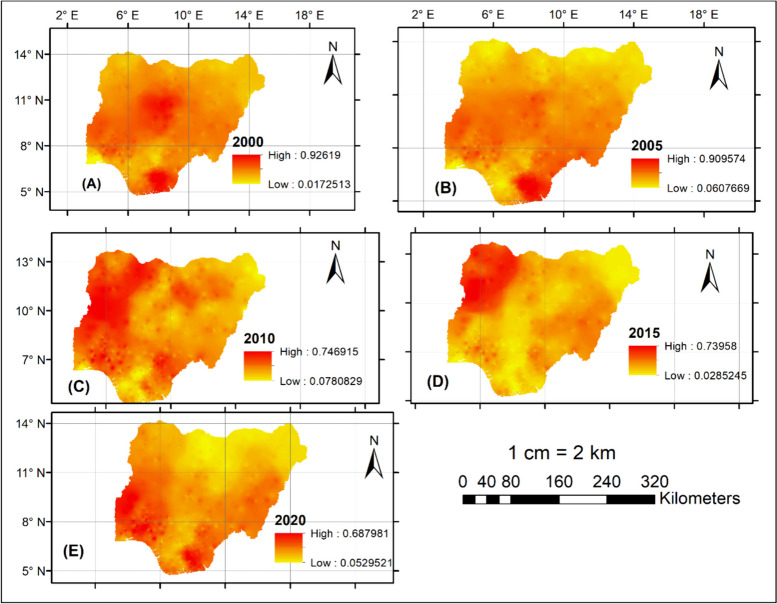
A five-year-period malaria frequency raster map for Nigeria during 2000 (**A**), 2005 (**B**), 2010 (**C**), 2015 (**D**), and 2020 (**E**)

Moreover, five malaria frequency raster maps were developed for the 5-year- intervals (Fig. 3A–E). Consequently, the appropriate spatial information has been well condensed, including geographic locations and spatial and temporal malaria changes. The most significant time frames were identified as 2000 (Fig. 4A) and 2020 (Fig. 4E); the highest (0.92619) and lowest (0.687981) frequencies were recorded, respectively. The raster maps also showed the center of malaria distribution in a portion of the southern region over time (Fig. 4A–E).

### Spatial distributions of malaria prevalence in 2000, 2005, 2010, 2015 and 2020

**Fig. 5:**
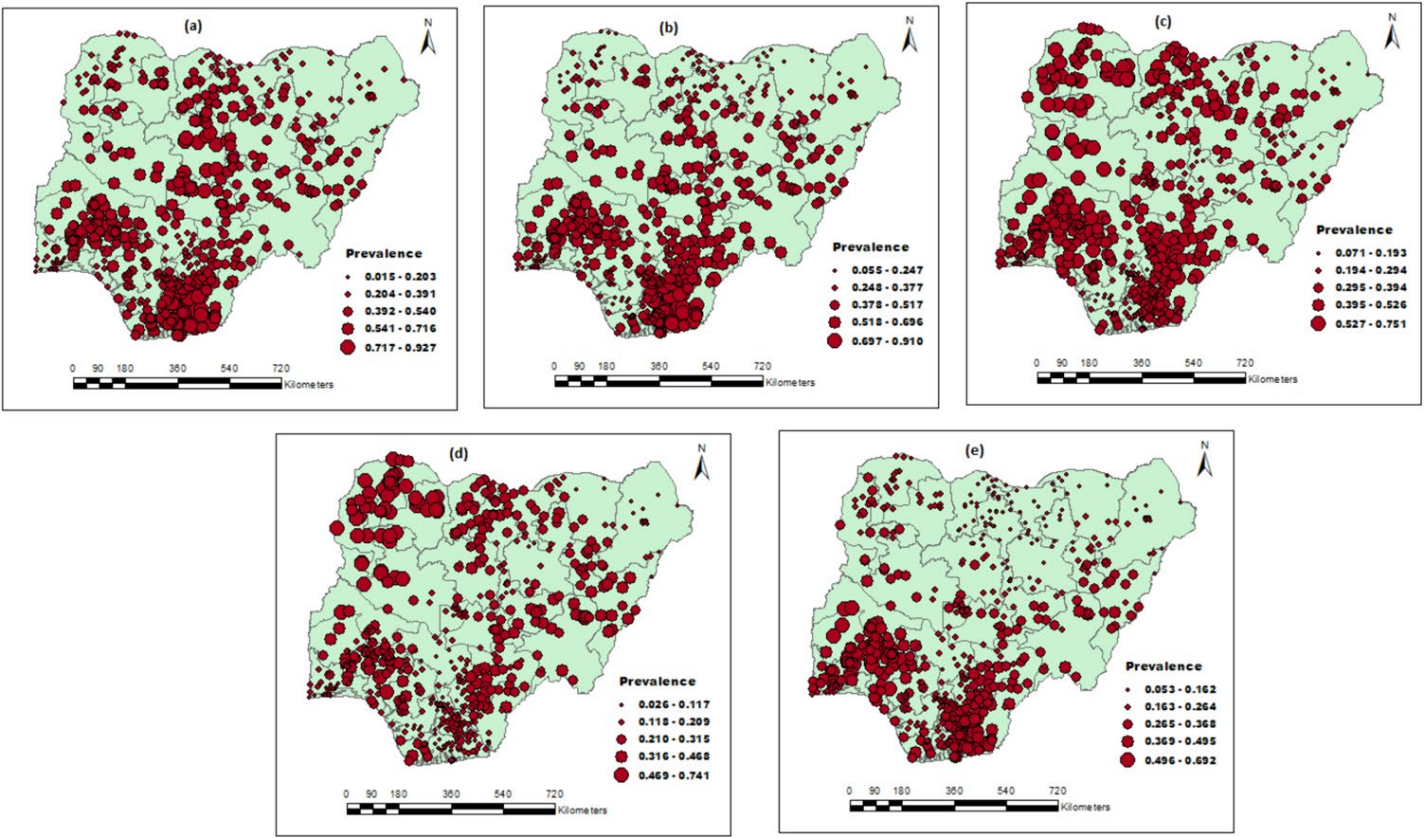
Geographical locations of data points and malaria prevalence in Nigeria: 2000(**a**), 2005(**b**), 2010(**c**), 2015(**d**), and 2020(**e**)

The detailed count of malaria cases in different regions is shown in Fig. 5. Spatial variations in malaria prevalence have been observed at regional levels. Malaria cases were concentrated in western, northwest, and eastern Nigeria. The lowest concentration was observed in Nigeria’s northcentral, northeastern, and south-south regions (Fig. 5).

**Fig. 6:**
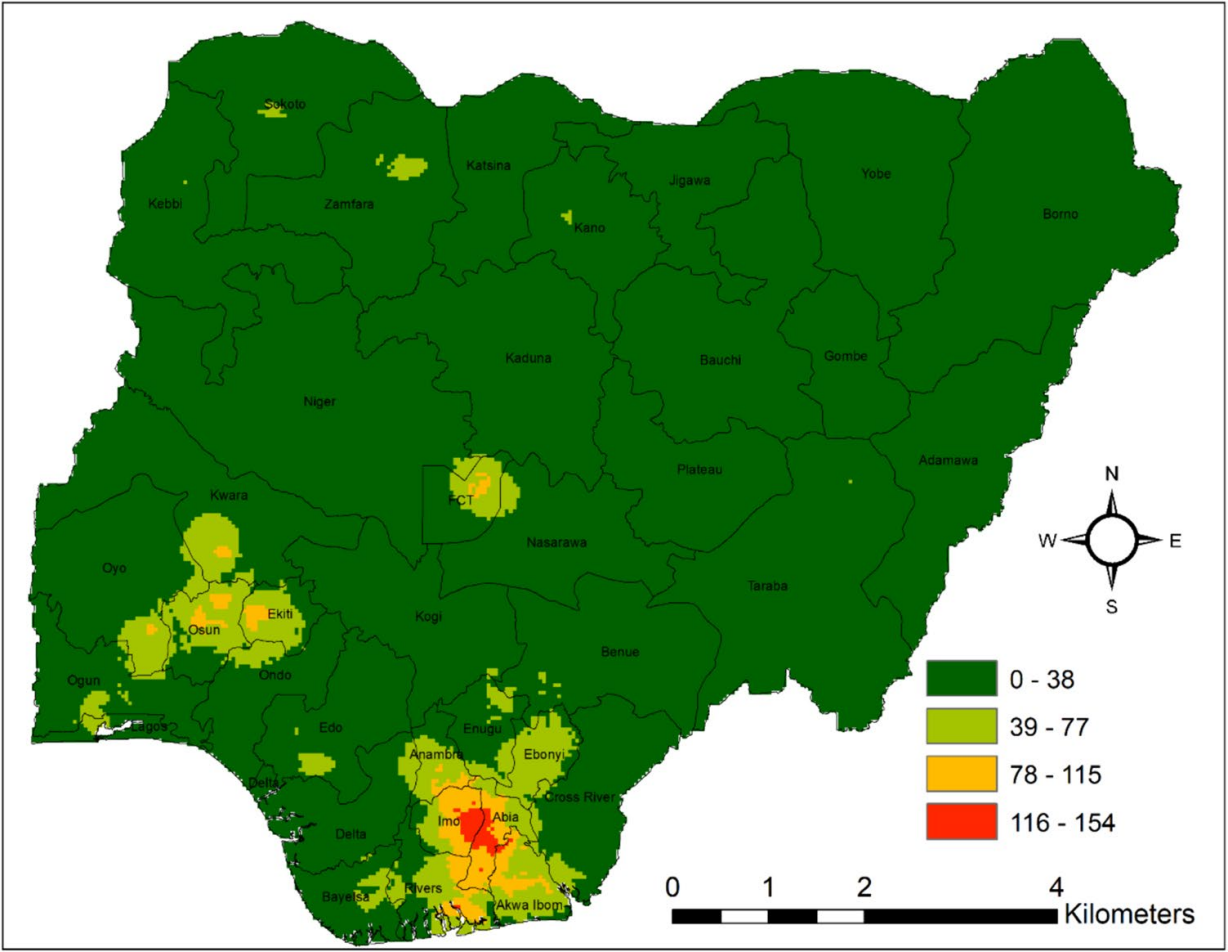
Point density map showing the distribution of malaria prevalence in the study areas from 2000–2020

Also, when considering the density map, high-density regions for malaria cases (between 77.12 and 154.24) at the 1-km spatial resolution, which is displayed in red and orange, have been located at the junctions between South-East (Imo and Abia State), and Southwest (Osun State) (Fig. 6).

### Spatial autocorrelation analysis (Moran’s I) of malaria

**Fig. 7:**
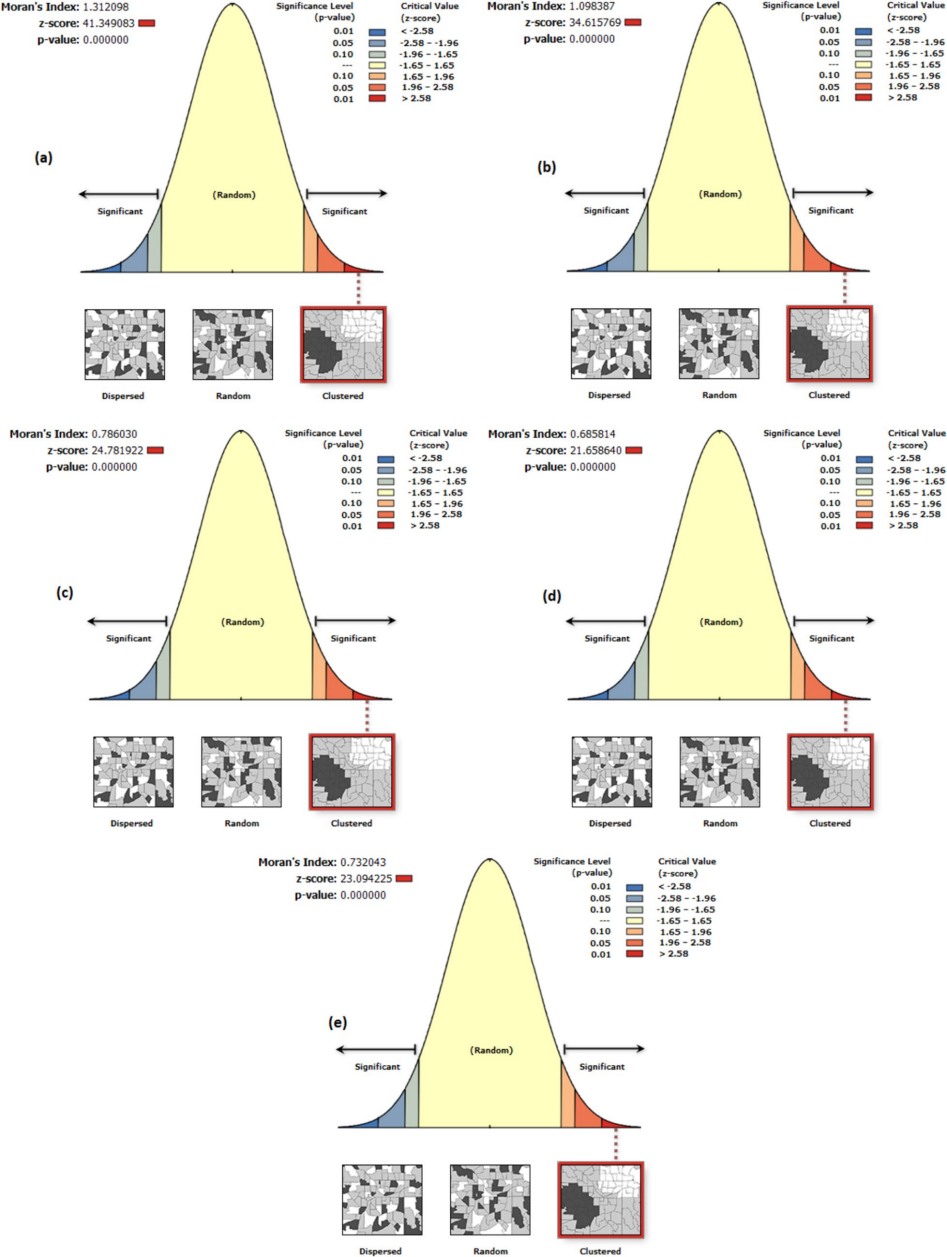
Spatial patterns of malaria prevalence in Nigeria: 2000(**a**), 2005(**b**), 2010(**c**), 2015(**d**), and 2020(**e**). The clustered patterns showed that the likelihood of occurrence by random chance is less than 1%

This section presents the results of methods used to analyze malaria cases. We used the Moran Global Index to estimate the overall degree of spatial autocorrelation. As Fig. 7 shows, the Moran Index value was positive, indicating statistically significant malaria within the study area. Global analysis of the spatial autocorrelation of individual surveys disclosed that there were substantial clustered trends of malaria across the country: Global Moran’s I = 1.312, Z-score = 41.35, *p*-value < 0.001 in NDHS 2000; Global Moran’s I = 1.098, Z-score = 34.62, *p*-value < 0.001 in NDHS 2005; Global Moran’s I = 0.786, Z-score = 24.78, *p*-value < 0.001 in NDHS 2010; Global Moran’s I = 0.686, Z-score = 21.66, *p*-value < 0.001 in NDHS 2015, and Global Moran’s I = 0.732, Z-score = 23.09, *p*-value < 0.001 in NDHS 2020 (Fig. 7a–e). In each output, the Z-score is primarily high and positive with a highly significant *p*-value, which showed 99% confidence for clustering malaria across Nigeria regions. The bright red (right side) and blue (left side) colors specified increased significance levels for which the probability of clustered patterns occurring by chance was less than 1%.

### Hot spot analysis of malaria prevalence between 2000–2020

**Fig. 8:**
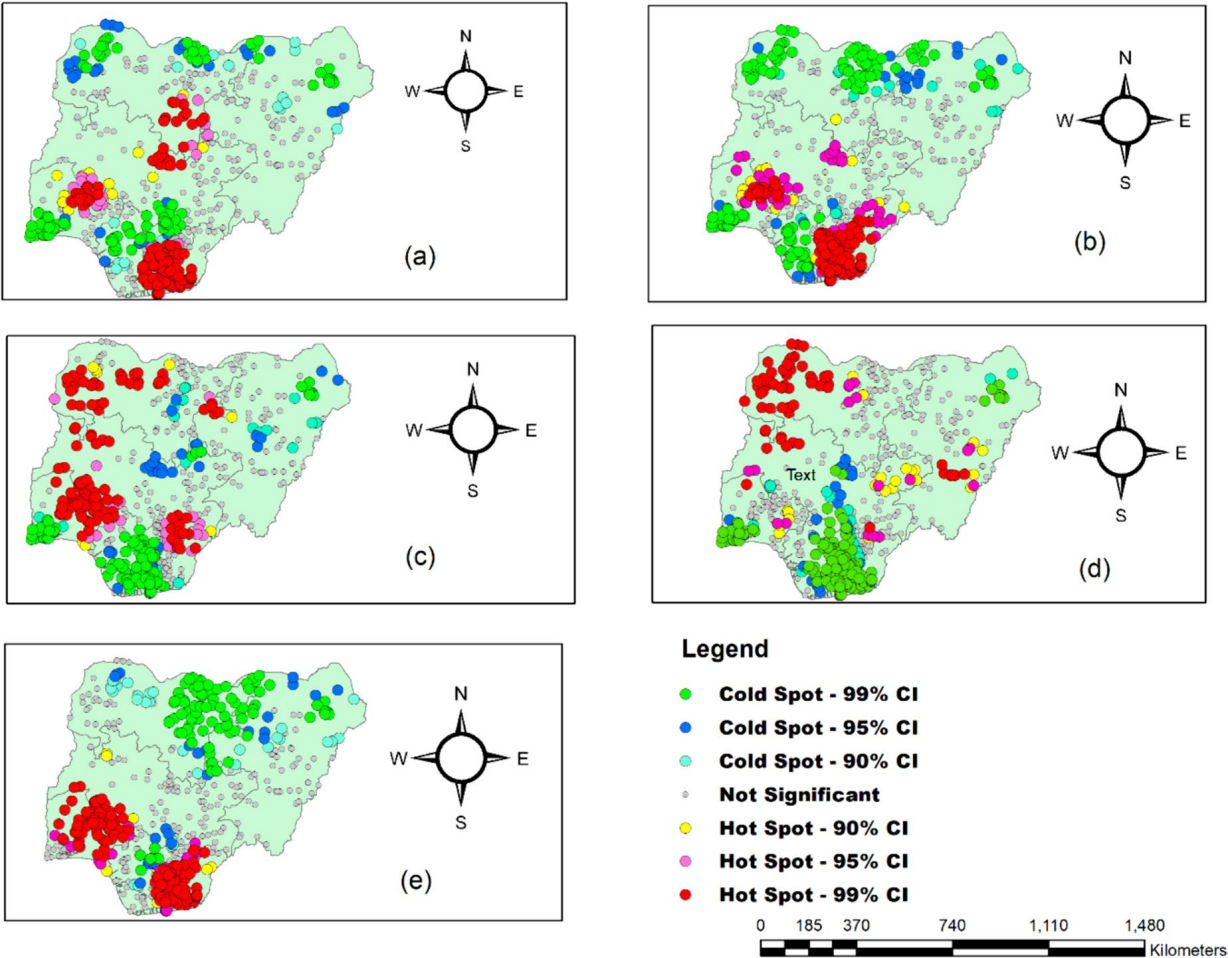
Hot spot analysis of malaria prevalence in Nigeria: 2000(**a**), 2005(**b**), 2010(**c**), 2015(**d**), and 2020(**e**)

Figure 8 presents the hot spot analysis using Getis-Ord Gi*. From Fig. 8a–d, red and yellow disclosed significant clusters of high-risk (hotspot) malaria zones, while green and blue disclosed substantial clusters of low-risk (cold spot areas). From the findings, in 2000, the hot spot zones of malaria prevalence were seen in parts of South-South, South-east, Southwest, Northcentral, and Northwest (Fig. 8a). On the other hand, in 2005, the hot spot zones of malaria prevalence were observed in regions of South-South, South-east, Southwest, Northcentral, while the cold spot zones were well pronounced in the Northeast, parts of the Northwest and South-South (Fig. 8b). Likewise, in 2010, the hot spot zones of malaria prevalence in Nigeria were identified in the Southwest, part of the Northwest, Northcentral, and Northeast, while the cold spot zones were more significant in South-South and part of Southwest regions (Fig. 8c). During NDHS 2015, statistically significant hot spot zones were seen in the Northern part of Nigeria. The statistically substantial cold spot zones were seen in the country’s southern region (Fig. 8d). Similarly, during NDHS 2020, statistically significant hot spot zones were seen in the southern part of Nigeria. The country’s Northern region saw statistically substantial cold spot zones, excluding North-Central (Fig. 8e).

## Discussion

Malaria is a severe menace to global health and is more critical in all regions of Nigeria, considering the country’s population. Previous findings on malaria modeling in Nigeria have reported higher malaria prevalence across various areas of Nigeria (Dawaki et al. 2016; Makinde et al. 2021; Beargie et al. 2019). The current study presents a spatiotemporal mapping of malaria prevalence and exploration of environmental inequalities in Nigeria for five years, ranging from 2000 to 2020. This study’s findings revealed that most malaria cases during the year investigated were substantially more extreme in the Northern region than in the Southern part of Nigeria. Conversely, the North Central region was more prevalent than all the Southern regions. However, the overall findings showed spatial discrepancies in the prevalence of the disease, indicating the northwest as the most affected area in the country. Also, the conclusions of this study identified the environmental indicators significant to malaria and determined their association with malaria prevalence using OLS regression. It was shown that enhanced vegetation index, annual land surface temperature, insecticide-treated bed net coverage, and mean temperature are significant indicators explaining the prevalence of malaria. Besides, precipitation also affects malaria prevalence in Nigeria. This is not surprising since variations in precipitation patterns in northern and southern Nigeria can affect the spread of malaria differently. An observation that suggests various parts of Nigeria may affect malaria diffusion differently. More precipitation occurs in southern Nigeria than in the Northern region. As a result, the spread is generally extreme early in the rainy and dry seasons.

The distribution of malaria spates shifted from the Northcentral and South-South region between 2000 and 2005 (Fig. 4A–B) towards the Northwest and Southwest region by the end of 2020 (Figs. 3C–E). However, the malaria distribution center turned to the Northern and part of the Southern region after 2005 (Fig. 3B), a finding that can be due to many reasons. One justification may be due to the movement from rural areas to the cities because of terrorist activities in some regions, and this may have contributed to the population increase within the towns in this region where the urban infrastructure became inadequate (Joshua et al. 2014; Eme et al. 2018). Besides, malaria cases had almost been eradicated in the Northeast region by the end of 2020 (Fig. 4E). This finding was consistent with the one conducted by Houben (Houben et al. 2013) in Northeastern Nigeria. The justification for these findings might align with the result of the Nigeria Malaria Indicator Survey 2021 (MIS), which established that mosquito nets are accessible to most of the states in the northeast region of Nigeria. For instance, the latest MIS report shows that bed nets are available in 68% of households in the Northeast of Adamawa (Nigeria Malaria Indicator Survey (NMIS) 2021).

Furthermore, the research findings indicated that environmental factors contribute to the increased malaria prevalence in the study area. A previous study by Sadoine et al. (2022) and Arhin et al. (2023) suggested that higher temperatures may lead to elevated malaria levels, potentially due to changes in mosquito populations. This underscores the importance of considering climate change in developing early warning systems and response strategies. Moreover, our study revealed similar positive correlations between malaria cases and environmental indicators. Supporting our findings, Tangena et al. (2023) reported a negative relationship between Insecticide-Treated Net Coverage (ITNC) and malaria cases. However, our results did not determine whether areas with high access to mosquito nets had a lower prevalence of malaria than areas with low access (Lindblade et al. 2015). Hence, this suggests that a cost-effective response program would consider promoting household access to local mass media, where household members receive frequent medical advice, particularly in rural areas with limited access to medical care.

Consequently, malaria prevalence was spatially clustered at the regional level during the separate investigation period of the study. The spatial distribution of the high prevalence of malaria during the respective survey period was in the western, northwest, and eastern parts of Nigeria. These variations could be due to climate change and declining precipitation distribution in these areas at different times. Spatial spreading has further revealed the spatial difference between malaria and other regions of Nigeria. For example, in the 2000 survey, statistically significant sensitive areas of malaria prevalence were found in the South-South, South-East, Southwest, Northcentral, and Northwest regions, while in the 2005 survey, malaria hotspots were observed in South-South, South-east, Southwest, Northcentral. This finding might be related to the variation in rainfall patterns. In 2011, the malaria hot spot was observed primarily in the Southwest, a portion of the northwest, Northcentral, and Northeast, while in the 2016 survey, statistically significant sensitive areas were observed in the northern parts of Nigeria (Ekpa et al. 2023). Likewise 2020, statistically substantial sensitive regions were found in southern Nigeria. This may be due to changing precipitation patterns in northern and southern Nigeria, which can affect malaria transmission dynamics differently (Okunlola and Oyeyemi 2019).

The study is limited by potential issues with data quality and gaps, particularly regarding the accuracy and accessibility of malaria cases and environmental data. While the temporal analysis might overlook short-term fluctuations in malaria rates, the regional spatial analysis could mask localized transmission patterns. Additionally, although the study identifies associations between environmental factors and malaria prevalence, it does not establish causation, thus limiting the generalizability of its findings to other locations or nations.

## Conclusion

This study utilized GIS to analyze malaria spatial patterns and environmental indicators across six regions in Nigeria. Malaria cases were concentrated in the western, northwest, and eastern areas, correlating positively with aridity, EVI, and precipitation. Despite an overall decline in malaria cases over time, particularly in the northwest region, there are still challenges in meeting the Sustainable Development Goal (SDG) target in the short term. Recommendations include focusing on rural areas with low socio-economic status and high malaria incidence, as well as scaling up interventions in areas with concentrated malaria prevalence. The study’s model equation, incorporating factors like EVI, ITNC, LST, mean temperature, and precipitation, provides evidence-based guidance for public health professionals and policymakers. Ultimately, the findings offer valuable statistical insights and inform policymaking decisions to address malaria and improve public health outcomes in Nigeria.

## Data Availability

Data supporting study findings are available for download from the DHS MEASURE website, conditional on approval from DHS, and will be made available upon request from the first author.
